# Can finite element models of ballooning procedures yield mechanical response of the cardiovascular site to overexpansion?

**DOI:** 10.1016/j.jbiomech.2016.06.021

**Published:** 2016-09-06

**Authors:** Giorgia M. Bosi, Benedetta Biffi, Giovanni Biglino, Valentina Lintas, Rod Jones, Spyros Tzamtzis, Gaetano Burriesci, Francesco Migliavacca, Sachin Khambadkone, Andrew M. Taylor, Silvia Schievano

**Affiliations:** a Centre for Cardiovascular Imaging, UCL Institute of Cardiovascular Science & Great Ormond Street Hospital for Children, London, UK; b Department of Medical Physics & Biomedical Engineering, UCL, London, UK; c Laboratory of Biological Structure Mechanics (LaBS), Chemistry, Materials and Chemical Engineering Department "Giulio Natta", Politecnico di Milano, Italy; d UCL Mechanical Engineering, Cardiovascular Engineering Laboratory, University College London, UK

**Keywords:** Sizing balloons, Finite element modelling, Implantation site mechanical response

## Abstract

Patient-specific numerical models could aid the decision-making process for percutaneous valve selection; in order to be fully informative, they should include patient-specific data of both anatomy and mechanics of the implantation site. This information can be derived from routine clinical imaging during the cardiac cycle, but data on the implantation site mechanical response to device expansion are not routinely available.

We aim to derive the implantation site response to overexpansion by monitoring pressure/dimensional changes during balloon sizing procedures and by applying a reverse engineering approach using a validated computational balloon model. This study presents the proof of concept for such computational framework tested *in-vitro*. A finite element (FE) model of a PTS-X405 sizing balloon (NuMed, Inc., USA) was created and validated against bench tests carried out on an *ad hoc* experimental apparatus: first on the balloon alone to replicate free expansion; second on the inflation of the balloon in a rapid prototyped cylinder with material deemed suitable for replicating pulmonary arteries in order to validate balloon/implantation site interaction algorithm.

Finally, the balloon was inflated inside a compliant rapid prototyped patient-specific right ventricular outflow tract to test the validity of the approach. The corresponding FE simulation was set up to iteratively infer the mechanical response of the anatomical model. The test in this simplified condition confirmed the feasibility of the proposed approach and the potential for this methodology to provide patient-specific information on mechanical response of the implantation site when overexpanded, ultimately for more realistic computational simulations in patient-specific settings.

## 1 Introduction

Percutaneous pulmonary valve implantation (PPVI) is an effective alternative to traditional open heart surgery for the treatment of pulmonary valve dysfunction (Cao et al., 2013, Hijazi et al., 2006, Lichtenstein et al., 2006, Lurz et al., 2008, Serruys, 2013, Walther et al., 2007, Zajarias and Cribier, 2009). Procedural success highly depends on patient selection and device positioning, in order to avoid embolisation on one hand, and overexpansion of the valve and vascular injury (i.e. arterial rupture, coronary compression) on the other (Fleming et al., 2012, Lurz et al., 2008, Shannon et al., 2011). This becomes even more important in patients with borderline dimensions and highly distensible implantation sites, the target for next generation of PPVI devices (Cao et al., 2014, Promphan et al., 2015, Schievano et al., 2010), where ballooning is used before device implantation to assess the mechanical response of the arterial wall and surrounding tissues to overexpansion (Boudjemline et al., 2012). A compliant sizing balloon is inflated in the right ventricular outflow tract (RVOT) under fluoroscopy guidance until parallel borders are achieved; the dimensions of the implantation site are captured at this point (Boudjemline et al., 2012). Following the indications of the sizing balloon, the experience of the interventional cardiologist indicates whether to perform or not the PPVI, but a proper quantification of the mechanical response of the RVOT and surrounding tissue is still lacking. Finite element (FE) simulations of the ballooning procedure could help gather further quantitative information on the interaction between the device and individual implantation sites during overexpansion (Bosi et al., 2015).

Several material models have been developed to take into account the non-linear, hyperelastic and anisotropic behaviour of arteries (Baek et al., 2007, Gasser et al., 2006, Holzapfel et al., 2000, Holzapfel and Gasser, 2001). Inverse methods to derive constitutive material parameters have been explored, combining inflation/extension experimental tests on animal (Badel et al., 2012, Flamini et al., 2015, Ning et al., 2010, Veljković et al., 2014) or human tissue (Avril et al., 2010, García-Herrera et al., 2013) with numerical analyses and optimisation methods. These *in-vitro* tests can provide the *ex-vivo* mechanical response of biological samples; however they neglect the *in-vivo* conditions, with pre-tensional state, presence of surrounding tissue (Kim et al., 2013), and dynamic effect of pulsatile flow (García-Herrera et al., 2013, Gasser et al., 2006, Holzapfel, 2006, O׳Dea and Nolan, 2012).

To overcome these limitations, inverse methods combining advanced image analysis (e.g. 4D echocardiography, computerised tomography and cardiac magnetic resonance) with numerical optimisation techniques have been explored to derive *in-vivo* patient-specific arterial wall constitutive material parameters. These non-invasive techniques are based on measured pressure and diameters during the cardiac cycle, and have been tested for animal (Smoljkić et al., 2015) and human arteries (Schulze-Bauer and Holzapfel, 2003), the latter being carotid (Masson et al., 2008) or aortic arteries (Karatolios et al., 2013, Wittek et al., 2013, Zeinali-Davarani et al., 2011). One of the first *in-vivo* studies correlated invasive pressure measurements with inner lumen diameter in order to obtain a two-dimensional Fung-type material model, able to capture non-linear and anisotropic response in the large strain domain (Schulze-Bauer and Holzapfel, 2003). Subsequently, one study employed dynamical intraluminal pressure measured by applanation tonometry, combined with medial diameter and intimal–medial thickness measured by high-resolution ultrasound echotracking, in order to iterativerly identify the material parameters for a non-linear, hyperelastic, anisotropic model (Masson et al., 2008). Alternatively, diastolic and systolic blood pressure measured in the brachial artery by sphygmomanometry combined with dimensional time resolved three dimensional ultrasound (4D echocardiography) imaging can also be used to reversely derive material properties (Wittek et al., 2013).

However, literature studies to date have focused on the determination of the mechanical behaviour during physiological loading conditions (i.e. during the cardiac cycle) and attempts to quantify the *in-vivo* mechanical response of the vascular site during overexpansion are lacking. By fully characterising and validating an FE model of the sizing balloon, and its expansion and interaction with the patient-specific arterial wall during sizing procedure, it would be possible to reverse engineer the patient-specific mechanical response and add this extra information to the implantation site constitutive curve.

Aim of this work is to present the proof of concept of such a computational framework, in a stepwise approach: first, the balloon FE model alone was validated by comparison with the balloon free expansion in an *ad hoc in-vitro* set-up; second, the balloon-vascular site interaction algorithm was validated by a simple experiment of balloon inflation in a compliant cylindrical phantom, previously characterised and modelled; finally, the FE framework was tested in a patient-specific phantom anatomy to add the geometrical complexity typical of PPVI patients and test the reverse engineering concept, in order to derive the implantation site mechanical response.

## 2 Materials and methods

A compliant sizing balloon PTS-X405 (NuMed, Inc., Hopkinton, NY, USA) with 40 mm diameter, 90 mm length and 0.5 atm (380 mm Hg) burst pressure was modelled in this study.

In order to replicate *in-vitro* the PPVI operating conditions and study balloon behaviour during inflation, an *ad hoc* experimental set-up was built in a catheterisation laboratory equipped with biplane fluoroscopy (AXIOM Artis, Siemens, Erlangen, Germany), as already described by Biffi et al. (2016). The bench unit included two bespoke holders to fix the balloon extremities and 4 metallic beads (0.5 mm diameter) placed on a central holder for image calibration. A 60 ml syringe pump (Graseby® 3200, Smiths Group, London, UK) filled with diluted contrast medium (1:1 in volume) was used to inflate the balloon at controlled constant flow rate (60 ml/h), while pressure and balloon profile were simultaneously recorded over time using a fibre-optic transducer (OPP-M250 OpSens Inc., Quebec City, Canada) and biplane x-ray images in the antero-posterior (AP) and lateral (LAT) projections. Balloon volume change during the inflation, V(t), was described as $V\left(t\right)=V_{0}+\dot{V}∙t$, where $V_{0}$ is the balloon initial volume, $\dot{V}$ the flow rate imposed by the syringe pump and t the time increment. To calculate $V_{0}$, the 2D fluoroscopy projections at zero pressure were calibrated and further elaborated by revolving the profile line of the balloon around its longitudinal axis (CAD software Rhinoceros, McNeel & Associates, USA). $V_{0}$ was calculated as the average between the volumes obtained from the AP and LAT projections. The balloon diameter was measured as the distance between the balloon contours in the central section, averaged between the AP and LAT projections.

The FE simulations of this study were performed in Abaqus/Explicit (Dassault Systèmes Simulia, Providence, RI, US) under the hypothesis of quasi-static conditions.

In order to compare *in-vitro* vs. *in-silico* results, the root-mean-square error between computational and experimental data was computed for pressure (${\mathrm{Err}}_{P}$) and diameter (${\mathrm{Err}}_{d}$) at equal inflation volumes.

### 2.1 Sizing balloon model

The sizing balloon FE model was designed based on the initial geometry from fluoroscopy pictures acquired at zero pressure during the free inflation experiment. Balloon material properties were obtained from uniaxial tensile tests performed on circumferential specimens from the central portion of the balloon. M3D4 membrane elements (3764 after sensitivity analysis) were chosen to discretise the model along with a hyperelastic third order Ogden energy function to describe the material:

$$U=\overset{N}{\underset{i=1}{\sum }}\frac{2\mu_{i}}{\alpha_{i}^{2}}\left(\lambda_{1}^{{-\alpha }_{i}}+\lambda_{2}^{{-\alpha }_{i}}+\lambda_{3}^{{-\alpha }_{i}}-3\right)+\overset{N}{\underset{i=1}{\sum }}\frac{1}{\delta_{i}}{({\text{J}}^{\mathrm{el}}-1)}^{2i}$$

where ${\bar{\lambda }}_{i}=J^{-1/3}\lambda_{i}$ are the deviatoric principal stretches, $\lambda_{i}$ are the principal stretches, *N* is a material parameter, *μ*_*i*_, *α*_*i*_ and *δ*_*i*_ are temperature-dependent material parameters, *J*^*el*^ is the elastic volume ratio (Table 1). The Poisson׳s ratio was assumed equal to 0.45 (Tzamtzis et al., 2013).

**Table 1:** Coefficients of the third order Ogden strain energy function for the sizing balloon material.

*i*	*μ_i_*	*α_i_*	*δ_i_*
1	−1183.29	−2.73	0.0056
2	470.70	−0.74	0
3	789.63	−4.96	0

**Fig. 1:**
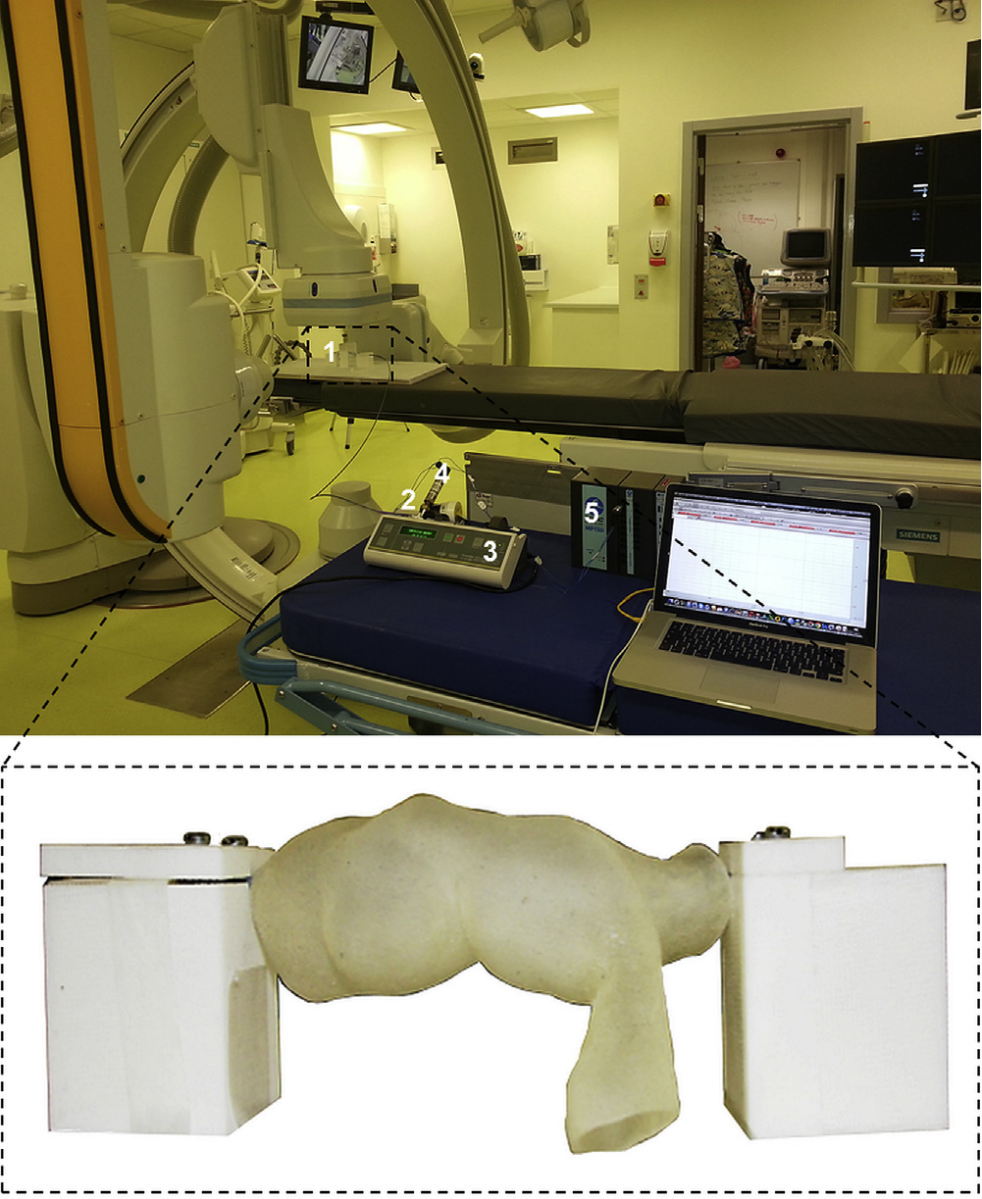
Experimental set-up for balloon inflation in the biplane fluoroscopy system catheterisation laboratory: (1) balloon, holders and calibration system, (2) 3-way valve, (3) syringe pump, (4) pressure transducer, (5) acquisition system. Magnification of the experimental set-up is shown for the patient-specific mock artery: purposely designed holders maintained fixed and suspended the balloon.

A balloon free inflation test was performed in the catheterisation laboratory set-up (Biffi et al., 2016, Cosentino et al., 2014) to derive pressure–volume (*P–V*) and diameter–volume (*d–V*) curves. The balloon was inflated up to the end of the syringe stroke, while pressure and x-ray images were simultaneously recorded (Fig. 1). The extremities of the FE balloon model were fixed in the axial and radial directions to reproduce the experimental conditions, and ‘fluid exchange’ and ‘fluid cavity’ functions were applied. The same inflation flow rate was imposed as in the *in-vitro* test, while *P* and *d* were set as output variables.

### 2.2 Cylinder model

A cylinder (thickness=1.2 mm, length=60 mm, diameter=20 mm) was 3D printed in the vertical orientation using TangoPlus FullCure^®^930 in the compliance range of pulmonary arteries (Biglino et al., 2013). Samples with same characteristics were 3D printed for tensile test assessment of the material mechanical behaviour in the circumferential direction.

A linear elastic and isotropic behaviour was assumed valid for the range of the deformation of interest. The material Young׳s modulus *E* was extrapolated from the tensile tests and applied to create the cylinder FE model. C3D8 8-noded hexahedral elements (~60,000) were used to mesh the cylinder.

A compliance test was performed by monitoring pressure variations while gradually increasing internal volume using syringes at both ends (Biglino et al., 2013), in order to assess distensibility (defined as $\text{D}=∆V/(∆P∙V_{o})$, with $V_{0}$=initial volume) and validate the corresponding FE cylinder model. The ends of the numerical model of the cylinder were fixed, thus simulating the presence of the syringes in the experimental tests, and the internal pressure measured throughout the experiment was applied to the inner surface of the cylinder. The FE model was validated by comparing the final internal volume.

### 2.3 Balloon-cylinder interaction

A new cylinder with the same material characteristics, but length=90 mm and diameter=28 mm, consistent with the clinical indications for use of PTS-X405 in pulmonary valve annuli, was integrated in the experimental set-up adopted for the free balloon expansion, in order to validate the sizing balloon model at lower volumes, as well as the interaction between the balloon and the simplified anatomical site. The balloon was inflated to the end of the syringe stroke. The FE models of balloon and cylinder were coupled as in the experiment, after a preliminary simulation carried out to crimp and deflate the sizing balloon, thus facilitating the insertion inside the cylinder. The balloon was virtually inflated by ‘fluid exchange’ with same flow rate as that used in the experimental test, and a general contact algorithm was set to allow the balloon-cylinder interaction. Computational *P–V* and *d–V* curves were extracted and compared to the experiment.

### 2.4 Patient-specific RVOT model

**Fig. 2:**
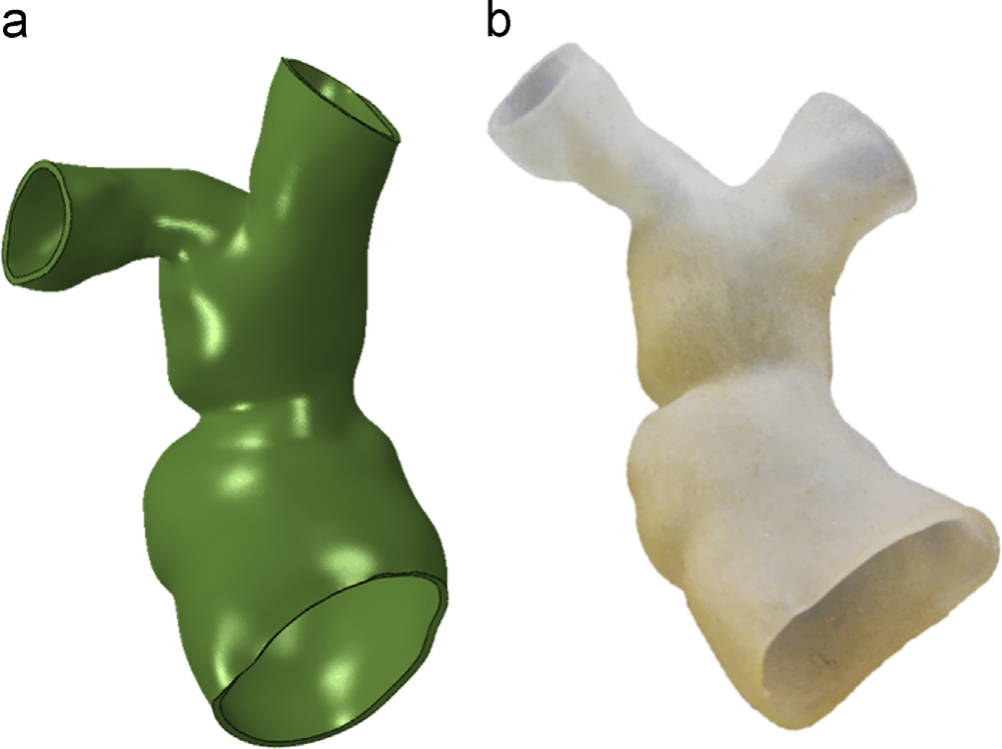
3D computer model (a) and corresponding rapid prototyping model (b) of patient-specific RVOT.

The RVOT blood pool of a PPVI candidate (17 year old, male) with free pulmonary regurgitation and borderline dimensions for available PPVI devices was reconstructed in 3D from cardiovascular magnetic resonance images (Fig. 2). The patient gave informed consent for use of his image data for research, as approved by the local Research Ethics Committee. The patient-specific anatomy was rapid prototyped with the same external thickness and material as the cylinder. The sizing balloon was inflated inside the anatomical model as done in clinical practice, and *P–V* and *d–V* curves were obtained. From such data, an initial Young׳s modulus (*E*_0_) was analytically estimated for the mock arterial wall using Laplace׳s law, assuming equivalence between the balloon pressure and the pressure on the vessel, and using the distance between the balloon borders at the level of the valve at the beginning (*P*=0 mm Hg) and end of the inflation test (maximum pressure). *E*_0_ does not represent the real Young Modulus of the mock implantation site, but only provides the starting point of the iterative method described here below.

The RVOT FE model was meshed with 259,278 C3D8 8-noded hexahedral elements, after sensitivity analysis. The inflation of the balloon inside the patient-specific geometry was replicated by applying the same procedure adopted for the balloon-cylinder system.

**Fig. 3:**
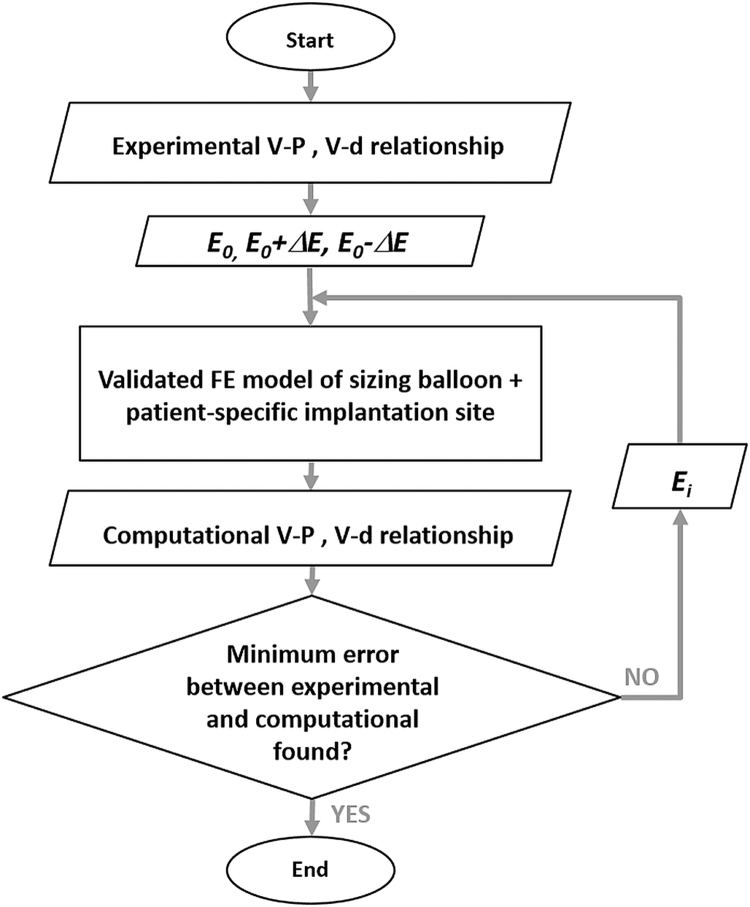
Flow chart of the iterative process used to infer RVOT mechanical response: an initial Young׳s modulus (*E*_*0*_) was derived from experimental *P–V* and *d–V* relationships. The FE model of sizing balloon inflated inside the patient-specific implantation site was fed with a range of Young׳s moduli in the neighbourhood of *E*_*0*_. The root mean square errors on pressure (*Err*_*P*_) and diameter (*Err*_*d*_) were calculated between computational and experimental curves. The process was iterated until the global minimum of the function *Err*_*P*_·*Err*_*d*_ was found.

An iterative method, schematised in Fig. 3, was set up to tune the Young׳s modulus of the wall, starting from the *E*_0_ analytically derived and feeding the FE model with a range of *E* values in the neighbourhood of *E*_0_, (*E*_0_±Δ*Ε*). The initial value Δ*Ε* was chosen equal to 0.20 MPa to narrow the range of confidence around the minimum, and then reduced to a value of 0.10 MPa, to obtain a more accurate value of the real *E*. The process was iterated until the global minimum of the error function *Err*_*P*_*·Err*_*d*_ was reached. The value of *E* resulting from the tuning process was then compared with the Young׳s modulus determined from the tensile test on the rapid prototyping material.

## 3 Results

The hypothesis of quasi-static condition was verified for all the implemented computational models.

**Fig. 4:**
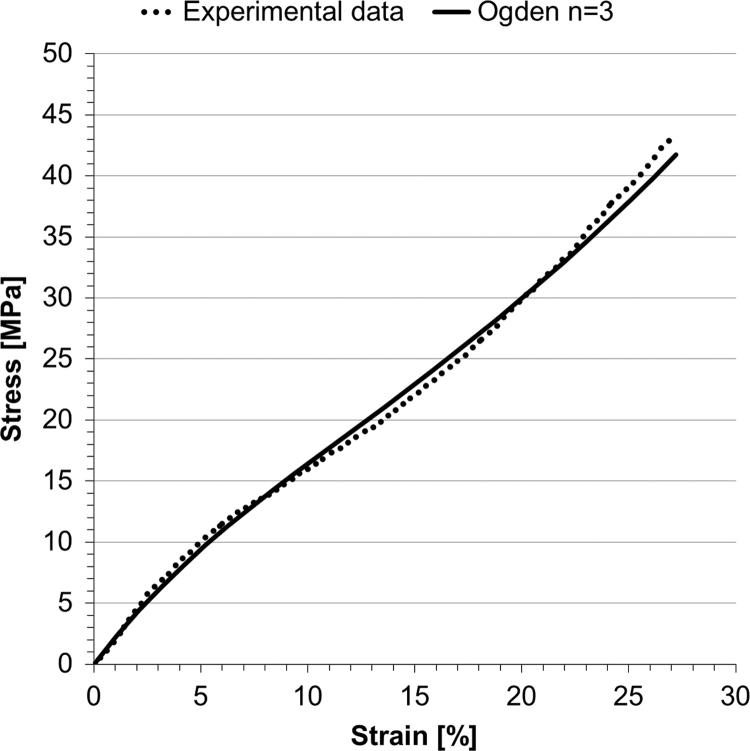
Stress–strain uniaxial tensile test for NuMed PTS-X405 sizing balloon, and data interpolated with a 3rd order Ogden energy function.

**Fig. 5:**
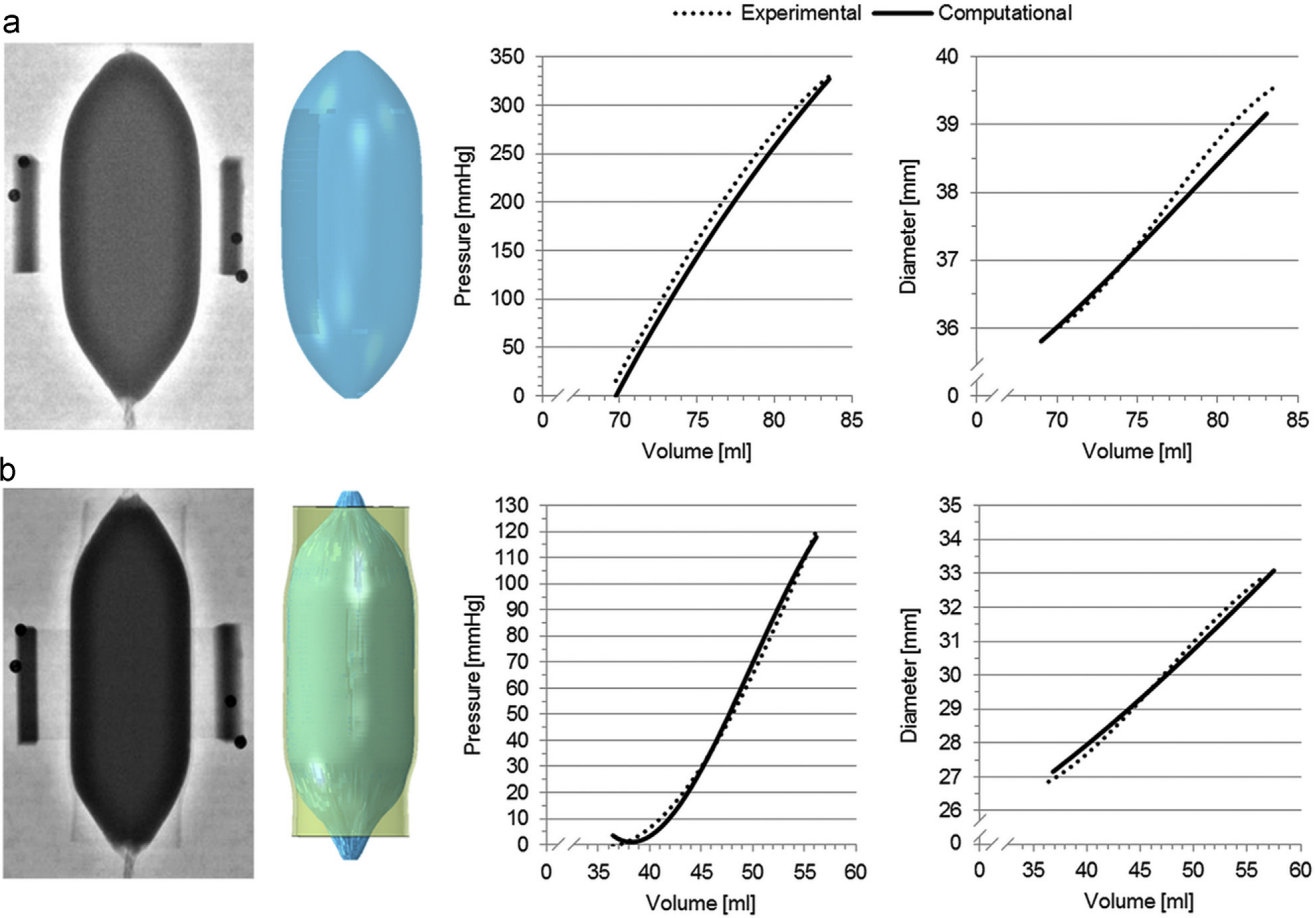
Comparison between the experimental (fluoroscopy images) and computational (3D shape) balloon inflations in the free expansion test (a) and in the interaction with the cylinder (b).

The stress–strain relationship from the uniaxial tensile test performed on the balloon material in the range of deformations of interest (*ε*<25%) is plotted in Fig. 4, and shows a non-linear hyperelastic behaviour. *V*_0_ for the free inflation test was 69.75 ml (*V*_0,*LAT*_=70.16 ml, *V*_0,*AP*_=69.35 ml) and the final volume was 83.55 ml, corresponding to a final pressure of 329.82 mm Hg, which is 51 mm Hg below burst pressure. The FE analysis modelled closely this behaviour (Fig. 5a, final volume of 83.07 ml for a final pressure of 326.98 mm Hg) with *Err*_*P*_=14.97 mm Hg and *Err*_*d*_=0.22 mm, the latter being below the fluoroscopy imaging system resolutions.

**Fig. 6:**
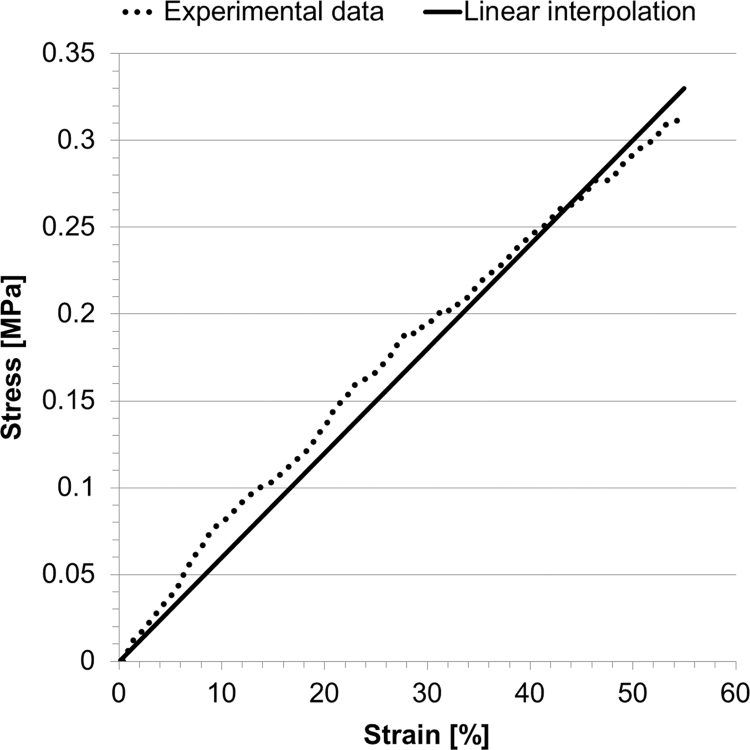
Uni-axial tensile test data from the rapid prototyping sample and their linear interpolation. The derived *E* was equal to 0.55 MPa.

The uni-axial test results for the rapid prototyping sample are shown in Fig. 6, leading to *E*=0.55 MPa. Final volume and pressure for the compliance test were respectively 17.18 ml and 99.24 mm Hg, thus yielding a distensibility equal to 6.4·10^−3^ mm Hg^−1^. Comparing computational results of cylinder deformations with the experimental counterpart yielded a volume error of 0.78% (17.05 ml vs. 17.18 ml). The cylinder material computational model can thus be considered validated and sufficiently robust to test it in more complex (i.e. realistic) geometric conditions.

*V*_0_ for the balloon-cylinder inflation test was 36.44 ml (*V*_0,*LAT*_=39.50 ml, *V*_0,*AP*_=33.37 ml) and the final volume was 56.18 ml for a final pressure of 120.45 mm Hg. The FE model produced a similar behaviour (Fig. 5b, final volume of 57.50 ml for a final pressure of 117.95 mm Hg) with *Err*_*P*_=2.95 mm Hg and *Err*_*d*_=0.21 mm, thus validating the interaction algorithm used.

**Fig. 7:**
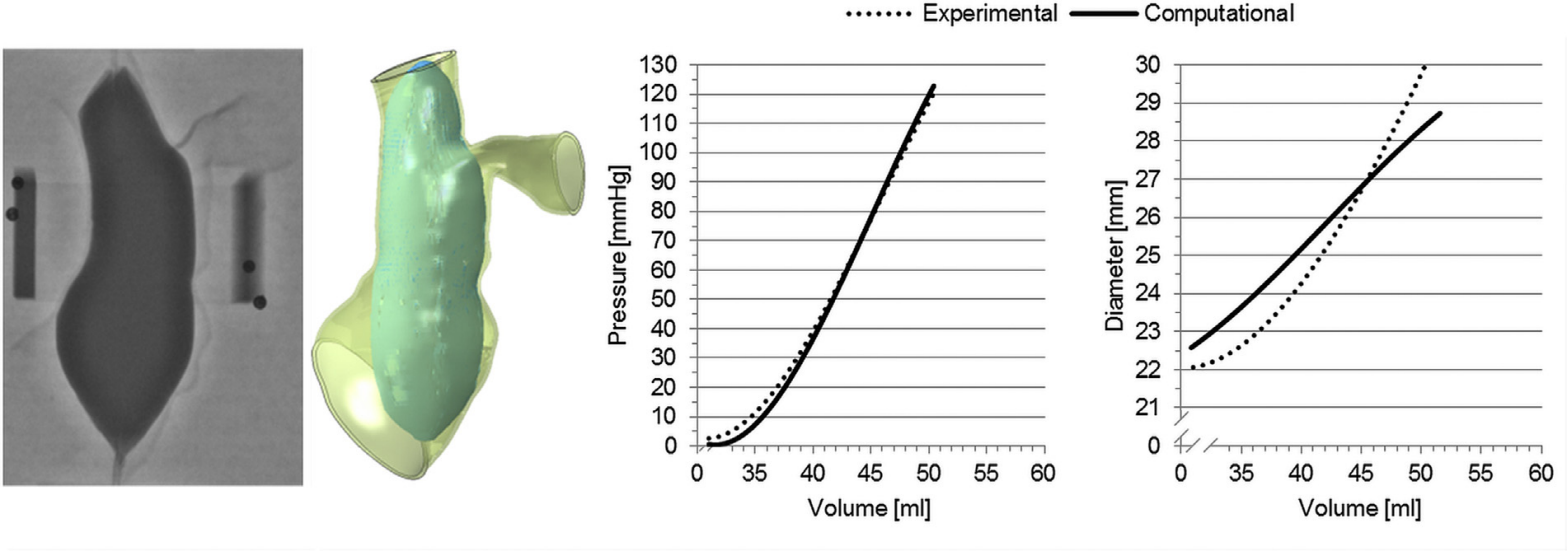
Comparison between the experimental and computational balloon inflation into the patient-specific implantation site.

**Fig. 8:**
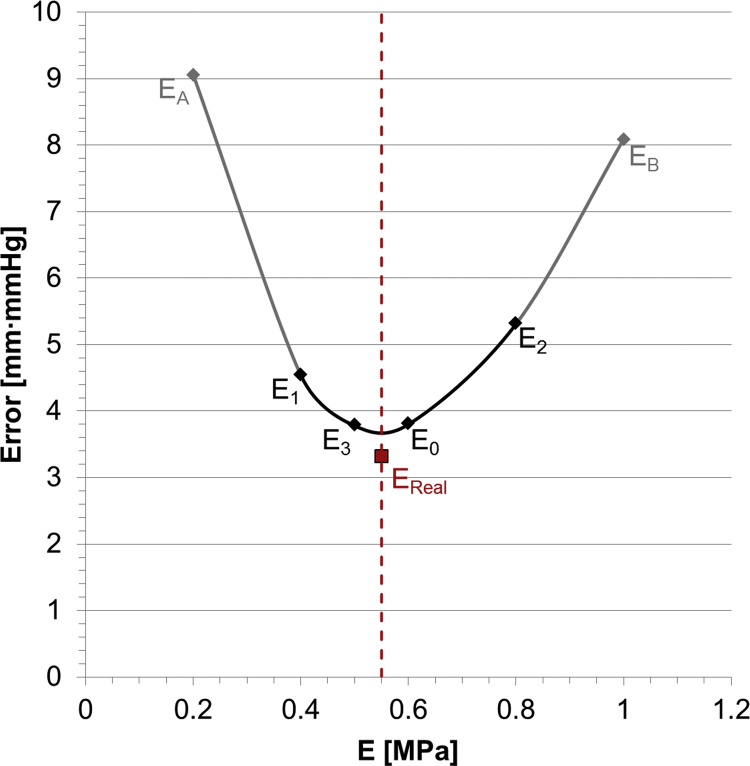
Error function obtained applying the iterative method to balloon/patient-specific anatomy: the minimum values were found for *E*=0.50÷0.60 MPa. The solid line interpolates the values found for the four iterations, while the dashed line represents the real Young׳s modulus (*E*_Real_=0.55 MPa) of the rapid prototyping material. Other two values are shown, corresponding to *E_A_*=0.20 MPa and *E_B_*=1.00 MPa, to confirm the robustness of the iterative method.

*V*_0_ for the balloon-RVOT interaction was 31.00 ml and the final volume of 50.44 ml equivalent to a final pressure of 120.39 mm Hg (Fig. 7). Minimum and maximum RVOT distances at the level of the valve were 22.61 mm (*d*_*min*,*LAT*_=21.96 mm, *d*_*min*,*AP*_=23.26 mm) and 29.96 mm (*d*_*max*,*LAT*_=28.14 mm, *d*_*max*,*AP*_=31.78 mm), respectively. *E*_0_ analytically estimated with Laplace law from the experiment inside the anatomical model was 0.60 MPa and four iterations were sufficient to reach the global minimum, specifically at *E*_1_=0.40 MPa, *E*_2_=0.80 MPa and *E*_3_=0.50 MPa. *Err*_*P*_ and *Err*_*d*_ were computed as function of the corresponding *E*, and the error function represented by their product is plotted in Fig. 8. Global minima were found in the neighbourhood of the Young׳s modulus experimentally determined from uniaxial tests on the TangoPlus FullCure^®^930 material, in particular *Err*_*P*_*·Err*_*d*_ was 3.78 mm·mm Hg for *E*_3_=0.50 MPa and 3.80 mm·mm Hg for *E_0_*=0.60 MPa. In Fig. 8, the error function is also plotted for the simulation corresponding to the real value of *E* used for manufacturing the RVOT was 0.55 MPa (*Err*_*P*_*·Err*_*d*_=3.31 mm·mm Hg*)*. Since the error function presents its minimum values in the neighbourhood of the real Young׳s modulus, we can conclude that the computational framework allowed us to satisfactorily assess the mechanical response of the surrounding structure in this patient-specific anatomy during overexpansion.

## 4 Discussion

Complex numerical models of isolated arteries which include patient-specific anatomical features, as well as sophisticated material models and properties derived from excised tissue mechanical tests have been described in the literature (Baek et al., 2007, Gasser et al., 2006, Holzapfel and Gasser, 2001, Taylor and Figueroa, 2009). However, patient-specific material characteristics have rarely been taken into consideration, due to the lack of methods to evaluate such properties *in-vivo*. Material properties derived from conventional mechanical tensile tests on *ex-vivo* arteries do not consider the *in-vivo* conditions and surrounding structures. Coupling of pressure information with dimension changes derived from patient image data can be used to infer mechanical information during the cardiac cycle (Karatolios et al., 2013, Masson et al., 2008, Schulze-Bauer and Holzapfel, 2003, Wittek et al., 2013, Zeinali-Davarani et al., 2011), but not at overexpansion. However, when deploying cardiovascular stents in the setting of distensible implantation sites, knowledge on the mechanical response to overexpansion could help cardiologists in the decision about the treatment strategy, and to guarantee safe positioning and anchoring of the device, avoiding the risk of vessel rupture.

In this study, we presented a computational framework that, coupled with measurements during sizing balloon procedures, could be used to quantify the patient-specific mechanical response to overexpansion *in-vivo* (Boudjemline et al., 2012). Simultaneous acquisitions of balloon inflation volumes, pressures and diameter changes during balloon sizing procedures under fluoroscopy guidance in the catheterisation laboratory can provide information not available otherwise through the conventional pre-procedural assessment of the patient.

The proposed method was tested as proof of concept in a mock arterial wall by comparison of the computational model of balloon-implantation site interaction with *in-vitro* data. The feasibility of this approach was proved in a stepwise manner: first, the sizing balloon was fully characterised and its finite element model validated against experimental data of free expansion. Then, a cylinder representing a simplified arterial wall was created and modelled to validate the numerical contact between the sizing balloon and the interacting structure. The cylinder was rapid prototyped in a material suitable for implementing realistic pulmonary artery distensibilities (Biglino et al., 2013). The computational simulations matched the equivalent experimental tests, thus validating the model of the balloon itself at lower volumes and the accuracy of the interaction algorithm with the idealised mock vessel. In a final test, an anatomical model was rapid prototyped using the same material as the cylinder, the balloon was inflated inside it and the validated balloon computational model was successfully used to infer the mechanical response during overexpansion as it would be done in clinical practice using the proposed methodology.

The mechanical response to overexpansion of the implantation site depends on many different factors including initial diagnosis, surgical history, cardiovascular conditions, and surrounding structures. Quantitative data about the mechanical response during overexpansion of different implantation sites (i.e. native, homograft, patch, calcified) through the proposed reverse engineering method could provide valuable information to assess PPVI candidates and to set more realistic patient-specific simulations for virtual device implantation, improving the decision-making process. Indeed, conversely to transcatheter aortic valve implantation (TAVI) where the implantation site is typically stiffened by calcifications, distensibility of the implantation site in PPVI, especially in the presence of native tissue, is a major determinant of the procedural success. Lack of quantifiable knowledge in terms of mechanical response during device expansion in such a variable population can result in less powerful prediction and lower confidence on the computational results when virtually simulating device implantation (Bosi et al., 2015).

The reverse engineering method here presented is a first step towards the estimation of the mechanical response to overexpansion of implantation sites from *in-vivo* data, presenting methodological considerations on the validation framework and showing promising results in the studied *in-vitro* case for the selected patient-specific anatomy and material. However, the robustness of the described approach will need to be proved by extending the study to a wider number of patient-specific geometries and different materials, first *ex-vivo* biological tissue (e.g. porcine arteries) and then explanted homograft/patches to assess diseased implantation sites; this would account for the complexity of non-homogeneous implantation sites.

The methodology here presented has been specifically designed to be employed, after further validation, in clinical applications, with the focus of quantifying the mechanical response to overexpansion of the patient-specific implantation site. It is important to note that the mechanical response during stent implantation is due not only to the arterial tissue itself, but also to the surrounding structures (Kim et al., 2013), and derived methods from *in-vivo* data cannot separate the two components. In this context, the simplest material model for the implantation site (i.e. homogeneous linear elastic) was preferred to more realistic and complex descriptions of arterial walls (heterogeneous, non-linear, anisotropic) (Baek et al., 2007, Gasser et al., 2006, Holzapfel and Gasser, 2001).

Lastly, the described method does not account for the pre-tensional stress and strains distribution from physiological loading condition. The model could be improved in the future by deriving the unloaded configuration of the implantation site through an inverse problem approach (Bols et al., 2013, Satriano et al., 2015, Vavourakis et al., 2016), thus restoring more realistic *in-vivo* stress and strain conditions.

The implemented balloon computational model was successful in replicating the *in-vitro* conditions in the range of tested deformations. However, when inflated inside patient-specific anatomies, the balloon unloaded configuration may need to be further analysed and improved, in order to better fit the balloon inside smaller implantation sites and to more realistically simulate the transitory inflation process. Uniform thickness membrane elements have been adopted for the balloon, but a more realistic varying thickness along the axial direction of the balloon could be implemented, even if this simplification did not seem to influence the good agreement of the computational model with the experimental data in our tested case. The balloon material has been tested only in the circumferential direction due to samples availability. Longitudinal samples should be tested to confirm isotropic behaviour in the range of deformation analysed. Lastly, cyclic loading tensile tests would allow quantification of the hysteretic properties of the balloon material, to better describe the balloon behaviour in case of repeated inflations. In addition, despite the proposed framework was developed and tested on a sizing compliant balloon, it could as well be adapted to non-compliant, high pressure balloons (Biffi et al., 2016), also employed in clinical practice to assess the landing zone for the current PPVI device and the risk of coronary compression.

Finally, in the validation process, the discrepancy on the diameter (*Err*_*d*_) measurement is likely to represent the main source of uncertainty of the proposed method, due to the complex morphology of the balloon at the end of inflation (when its shape adapts to the implantation site anatomy) that makes diameter measurements possibly inaccurate. To account for this, different weights could be assigned to *Err*_*p*_, *Err*_*d*_ when computing the *Err* function.

## 5 Conclusions

In this study, we have introduced a new approach to infer the mechanical response of the implantation site during cardiovascular stent implantation and we successfully tested the proposed method as proof of concept on a rapid prototyped anatomy of a patient right ventricular outflow tract. The implemented reverse engineering method, combined with patient-specific information during balloon sizing procedures, can enable the acquisition of *in-vivo* data currently not available, improving substantially the patient-specific modelling of the device-implantation site.

## Conflicts of interest

The authors report no conflicts of interest.
